# Advances in transposable elements: from mechanisms to applications in mammalian genomics

**DOI:** 10.3389/fgene.2023.1290146

**Published:** 2023-11-30

**Authors:** Mei Han, Matthew H. Perkins, Leonardo Santana Novaes, Tao Xu, Hao Chang

**Affiliations:** ^1^ Guangzhou National Laboratory, Guangzhou, China; ^2^ Nash Family Department of Neuroscience, Icahn School of Medicine at Mount Sinai, New York, NY, United States

**Keywords:** transposable elements, tumor genetics, aging, immune response, forward genetic screening, high throughput sequencing, RNA-guided transposition

## Abstract

It has been 70 years since Barbara McClintock discovered transposable elements (TE), and the mechanistic studies and functional applications of transposable elements have been at the forefront of life science research. As an essential part of the genome, TEs have been discovered in most species of prokaryotes and eukaryotes, and the relative proportion of the total genetic sequence they comprise gradually increases with the expansion of the genome. In humans, TEs account for about 40% of the genome and are deeply involved in gene regulation, chromosome structure maintenance, inflammatory response, and the etiology of genetic and non-genetic diseases. In-depth functional studies of TEs in mammalian cells and the human body have led to a greater understanding of these fundamental biological processes. At the same time, as a potent mutagen and efficient genome editing tool, TEs have been transformed into biological tools critical for developing new techniques. By controlling the random insertion of TEs into the genome to change the phenotype in cells and model organisms, critical proteins of many diseases have been systematically identified. Exploiting the TE’s highly efficient *in vitro* insertion activity has driven the development of cutting-edge sequencing technologies. Recently, a new technology combining CRISPR with TEs was reported, which provides a novel targeted insertion system to both academia and industry. We suggest that interrogating biological processes that generally depend on the actions of TEs with TEs-derived genetic tools is a very efficient strategy. For example, excessive activation of TEs is an essential factor in the occurrence of cancer in humans. As potent mutagens, TEs have also been used to unravel the key regulatory elements and mechanisms of carcinogenesis. Through this review, we aim to effectively combine the traditional views of TEs with recent research progress, systematically link the mechanistic discoveries of TEs with the technological developments of TE-based tools, and provide a comprehensive approach and understanding for researchers in different fields.

## Graphical Abstract

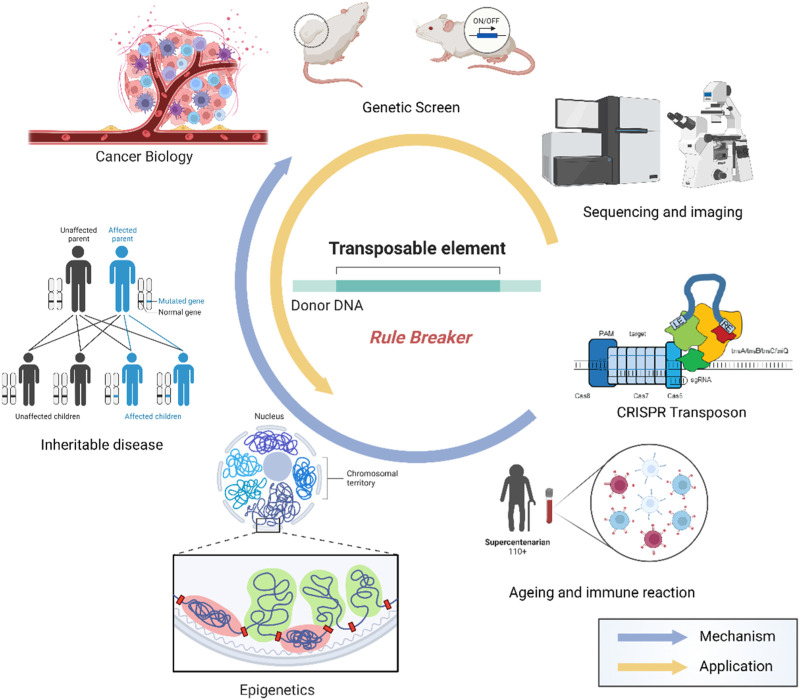

## 1 Introduction

Transposable elements (TEs) are mobile DNA sequences that mainly range in length from 100 bp to 10,000 bp, and the dynamic features of their interaction with host genomes drive evolutionary novelty and shape genome compositions and functions in multiple ways. TEs were found to exist in all organisms throughout prokaryotic and eukaryotic kingdoms. TEs-derived sequences constitute a large portion of most eukaryotic genomes, which comprise approximately 10% of some fish genomes, 37% of the mouse genome, 45% of the human genome, and more than 90% of some plant genomes. An interesting discovery is that the genome sizes of different species are linearly related to the portion of its contents. Similar to viruses, TEs are multi-faceted selfish genetic elements that encode and regulate proteins with multiplexed functions and noncoding regulatory elements by replication or remobilization themselves. To differentiate them from other invasive genetic elements like viruses, a TE is defined as a genetic element with the potential for chromosomal and replicative mobilization, and they exhibit an increasing frequency of vertical transmission through the germline.

**FIGURE 1:**
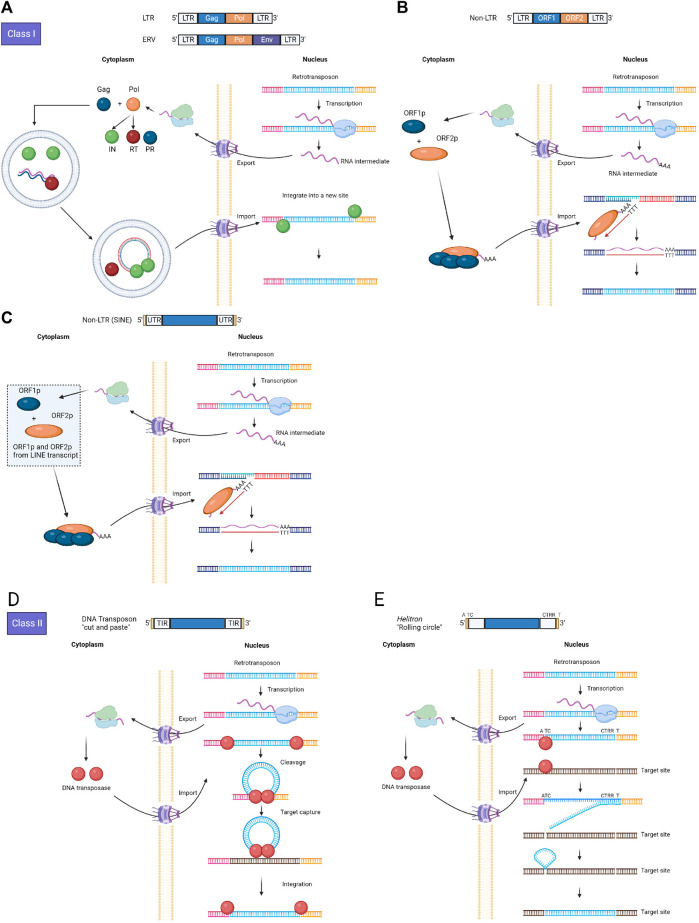
Summary of replication mechanisms of Class I and Class II TE subtypes. **(A)** Simplified representations of major steps in the transposition cycles of LTR and ERV; **(B)** Simplified representations of major steps in the transposition cycles of Non-LTR (LINE); **(C)** Simplified representations of major steps in the transposition cycles of Non-LTR (SINE); **(D)** Simplified representations of major steps in the transposition cycles of DNA transposon (“cut-and-paste” model); **(E)** Simplified representations of major steps in the transposition cycles of DNA transposon (“Rolling circle” model). Abbreviations: LTR, long terminal repeats; ERV, Endogenous retroviruses; LINE, long interspersed nuclear elements; SINE, short interspersed nuclear elements; IN, integrase; PR, protease; RT, reverse transcriptase; Pol, polyprotein; UTR, untranslated regions; TIR, terminal inverted repeat.

In the decades following Barbara McClintock’s groundbreaking discovery of mobile DNA sequences, the profound impact of TEs on both evolution and human health has become evident. To understand how TEs have impacted biological functions, we have to understand the diversity, classification, and transposition mechanism of TEs. TEs can be categorized into two major classes based on their transposition intermediates: class I, retrotransposons, and class II, DNA transposons (Figure 1) (Wicker et al., 2007). Class I TEs replicate based on the copy-and-paste mechanism that needs two stages. First, they are transcribed from DNA to RNA. Then, the intermediate RNA is reverse-transcribed to DNA, and this copied DNA sequence is inserted back into a new genome position. Both the reverse transcriptase and the transposase are usually involved in this process. There are three main types of retrotransposons: a) long terminal repeat (LTR) elements, b) Long interspersed nuclear elements (LINEs) which encode reverse transcriptase but lack LTR elements, and c) Short interspersed nuclear elements (SINEs) which do not encode reverse transcriptase (Figures 1A–C). The highest abundance of TEs in human genomes is one form of the non-autonomous SINEs, the Alu repeats (∼10.6%), and one form of the autonomous LINEs, the LINE-1 or human L1 repeats (∼16.9%), both of which belong to the non-LTR retrotransposons.

In contrast, class II TEs are usually mobilized in the genome based on a cut-and-paste mechanism, in which a DNA transposon is excised and moved to a new location of the genome by itself with the catalysis of a DNA transposase. At present, there are four main classifications based on the remobilization mechanism for DNA transposons: a) “cut and paste” elements mobilized by DDE-transposase (DDE-transposases belong to a large superfamily of polynucleotidyl transferases, including RNase H, RAG proteins, and retroviral integrases); b) “cut and paste” elements mobilized using tyrosine recombinase (YR) (known as Cryptons); c) “rolling-circle” elements (known as Helitrons); and d) “self-synthesizing” elements (called Polintons) (Figure 1D). Compared with retrotransposons, DNA transposons are much less abundant in the human genome, accounting for 2.8% of the entire genome and 6% of all TEs. Human DNA transposons can be classified into seven major groups, encompassing i) Charlie, ii) Zaphod, iii) Tc2, iv) PiggyBac-like, v) Mariner, vi) Tigger, and vii) unclassified elements (Lander et al., 2001).

This review is organized into two parts; we first discuss recently discovered mechanisms by which TEs contribute to disease and healthy aging. TEs’ random insertions contribute to heritable diseases and tumorigenesis, with over 100 such conditions attributed to transposition mutagenesis. Germline insertions with overt influences on nearby gene functions can lead to several monogenic diseases and increase the risk of complex diseases if the mutants spread in numerous populations. Meanwhile, somatic retrotransposition and chromosome rearrangement induced by altered TEs expression have been shown in many malignant tumor formations. In the second part, we look at the recent advances in the applications of TEs for mutagenesis and molecular biology applications. As a powerful genetic tool, TEs have been modified and employed to decipher biological functions. These novel applications help us to systematically discover a series of oncogenic and tumor suppressor genes in many types of cancers and phenotypic disorders. As an efficient insertional tool, TEs have been used to integrate barcodes, primer sequences, or other fluorescent dye-labeled sequences into genome DNA *in vivo* or *in vitro*, providing an influential strategy for high-throughput sequencing library preparation or high-resolution imaging. In this review, we offer a comprehensive guide to TEs in molecular genetics, human health, and biotechnology. For an in-depth review of classification, evolution, and applications in lower-level organisms like yeast, fruit flies, and plants, we recommend these other reviews (Feschotte et al., 2002; Bessereau, 2006; Bleykasten-Grosshans and Neuveglise, 2011; Rebollo et al., 2012; Merel et al., 2020; Moschetti et al., 2020; Wells and Feschotte, 2020).

## 2 TEs in human health and diseases

### 2.1 Involvement of TEs in human cancer

Genomic rearrangements are a hallmark of human cancers (Hanahan and Weinberg, 2011), with somatic mutagenesis induced by LINE-1 insertions being a prevalent occurrence across many cancer types (Lee et al., 2012; Xiao-Jie et al., 2016). One of the essential roles of TEs in carcinogenesis is disrupting expression levels of tumor suppressor genes. In 1988, a case of LINE-1 insertion into the c-myc gene was described by Morse and colleagues in a breast cancer sample (Morse et al., 1988). In 1992, Nakamura and colleagues reported on a case of colorectal cancer in a patient where the coding exon of the adenomatous polyposis coli (APC) gene was disrupted by a 750-bp LINE-1 insertion (Miki et al., 1992). New evidence indicates that LINE-1 transposition activity confers a high risk of incidence and a poor survival rate of APC-related colon cancer (Scott et al., 2016; Cajuso et al., 2019). Other tumor suppressor genes that have been reported to be mediated by hyperactivation of LINE-1 include RB1, FSTL5, and FGGY, which are finally considered to promote the development and progression of retinoblastoma, pancreatic ductal adenocarcinoma, and lung squamous cell carcinoma in patients (Rodic et al., 2015; Rodriguez-Martin et al., 2016; Zhang et al., 2019). The non-autonomous Alu element has been identified to be inserted in the BRCA2 gene, resulting in the removal of a targeted exon from the corresponding mRNA molecule, which is involved in the development of breast cancer (Teugels et al., 2005). Hyperactivation and high mobilization of TEs are mainly caused by methylation loss. Moreover, hypomethylation in a specific promoter region of LINE-1 near the intron 19 of oncogene anaplastic lymphoma kinase (ALK) can alter transcription initiation and lead to overexpression of a specific form of ALK. This new form of ALK was detected in 11% of melanoma samples (Wiesner et al., 2015).

Here, we will delve into the latest breakthroughs in foundational subjects, addressing key questions such as: a) Can the hyperactivation of transposable elements serve as a diagnostic marker for human cancer? b) When do somatic LINE-1 insertions become influential in tumorigenesis and cancer progression? c) Are there emerging mechanisms, like DNA transposons, that play a role in tumorigenesis as well?

#### 2.1.1 TE, tumor types, endogenous tumor regulator, and diagnostic marker

**FIGURE 2:**
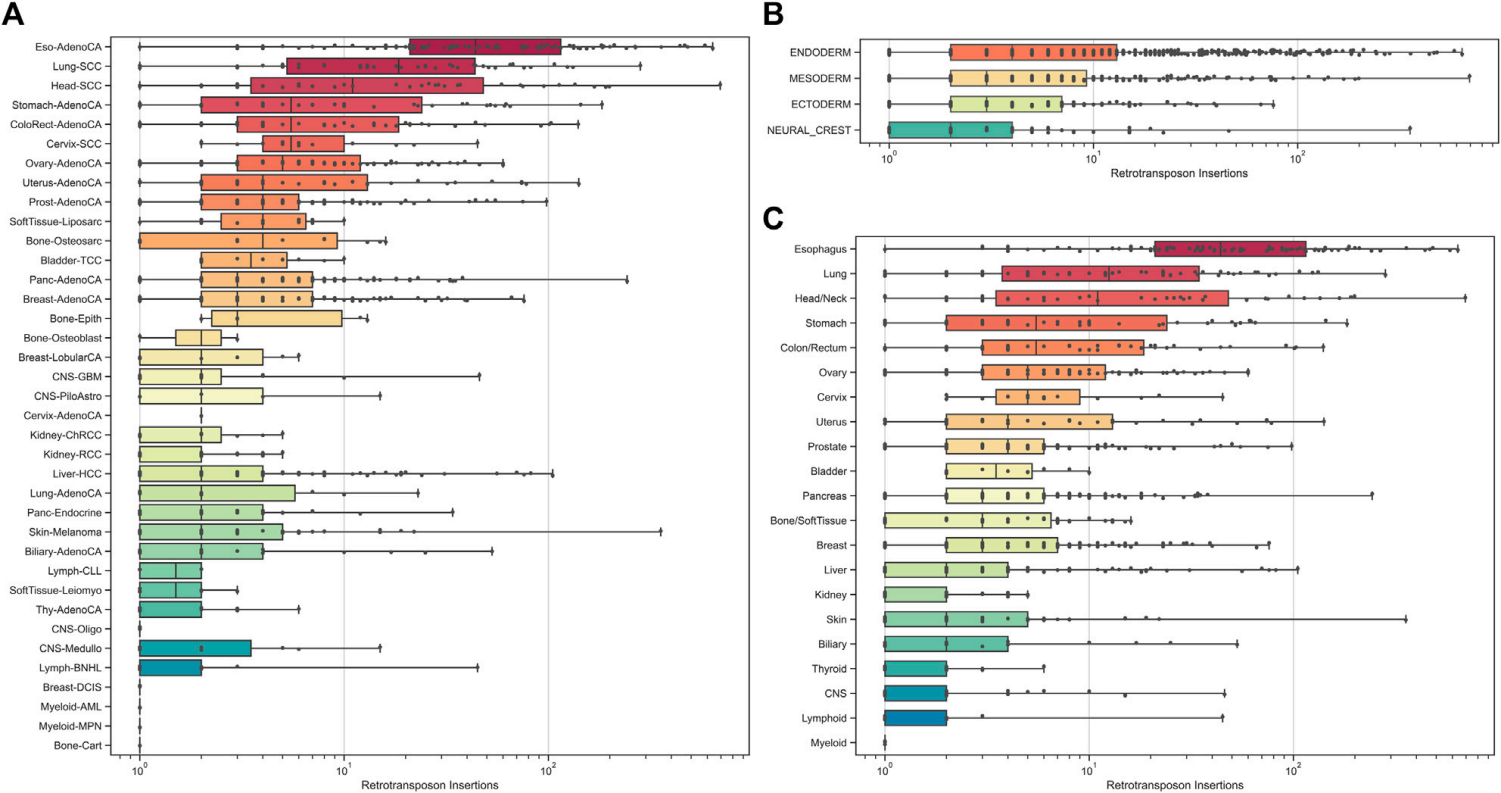
Distribution of somatic retrotransposon insertions (data reanalyzed from ref36). **(A)** Distribution of somatic retrotransposon insertions of different classes across tumor types; **(B)** Distribution of somatic retrotransposon insertions of different tumorigenesis germ layers; **(C)** Distribution of somatic retrotransposon insertions of different tumorigenesis organ types. Retrotranspositions are counted by summing somatic retrotransposon insertions, transductions, and somatic pseudogene insertions.

Nearly half of human cancers exhibit abnormal levels of somatic LINE-1 retrotransposition (Lee et al., 2012; Solyom et al., 2012; Helman et al., 2014; Tubio et al., 2014). A recent study confirmed that L1 integration dominates the landscape of somatic retrotransposition in the PCAWG dataset, which comprises 98% of total events (18,739 out of 19,166). By contrast, Alu and SVA represent the minor categories (130 and 23 of 19,166). When we look at tumor types, LINE-1 integrations are one of the earliest and most reported in the gastrointestinal tract, including colorectal cancer, esophageal adenocarcinomas, hepatocellular carcinomas (Shukla et al., 2013), and pancreatic ductal adenocarcinoma (Ewing et al., 2015; Rodic et al., 2015). Additionally, head and neck cancers (Helman et al., 2014; Tubio et al., 2014) and prostate and ovarian cancers (Lee et al., 2012; Tubio et al., 2014) are also frequently shown to be acquired by LINE-1 insertions. Through a comprehensive analysis of 2,954 cancer genomes spanning 38 histological cancer subtypes, significant increases in somatic retrotransposition were observed in esophageal adenocarcinoma, head-and-neck squamous carcinoma, lung squamous carcinoma, and colorectal adenocarcinoma (ICGC/TCGA Pan-Cancer Analysis of Whole Genomes Consortium, 2020). Notably, esophageal adenocarcinoma exhibited the highest incidence of LINE-1 retrotransposition (ICGC/TCGA Pan-Cancer Analysis of Whole Genomes Consortium, 2020). In contrast, acute myeloid leukemias, plasma cell myelomas, and high-grade gliomas show little evidence of association with alterations in somatic retrotransposition (Figure 2).

Endogenous TE can offer many regulatory sequences contributing to upstream and downstream gene expression by functioning as enhancers, promoters, silencers, and boundary elements (Moschetti et al., 2020; Sundaram and Wysocka, 2020). Unbiased, genome-wide measurements of episomal enhancer activity using the complete human genome library and comprehensive epigenetic profiling were employed to define TE-derived enhancers in colorectal and hepatocellular carcinomas (Karttunen et al., 2023). The findings indicate that TEs can function as tissue-specific enhancers, impacting differential gene activity in human cancers.

This raises a question: Is LINE-1 a viable diagnostic biomarker for early cancer detection? There are two essential proteins associated with LINE-1: ORF1p and ORF2p. ORF1p encodes an RNA-binding protein that forms trimers to bind single-strain RNA, and ORF2p consists of an endonuclease domain and a reverse transcriptase domain, which play a critical role in LINE-1 remobilization. The expression level of ORF1p is much higher than ORF2p, and it is considered a hallmark of human cancers, especially for breast, ovarian, and pancreatic cancers (Leibold et al., 1990; Rodic et al., 2014; Rodic et al., 2015; Ardeljan et al., 2017). A study examined ORF1p immunolabeling on 1,027 cases of human neoplasms and found that 47% of these were immunoreactive (Rodic et al., 2014). This study also indicates that LINE-1 ORF1p overexpression cancers were more commonly TP53 deficient (Rodic et al., 2014). When considering the primary site of origin, ORF1p was detectable in 97% of breast cancers, 93.5% of ovarian cancers, 89% of pancreatic cancers, and almost 60% of the endometrium, biliary tract, esophagus, bladder, head and neck, lung, and colon cancers (Rodic et al., 2014). In contrast, cancers in the kidney, liver, cervix, and prostate were infrequently expressed ORF1p (24% in mean) (Rodic et al., 2014). Another interesting study shows that 74% of secondary glioblastomas express ORF1p, a proportion almost two-fold higher than primary glioblastomas. Paradoxically, LINE-1 retrotransposition plays little role in either primary or secondary glioblastomas (Rodic et al., 2014; Achanta et al., 2016).

LINE-1 ORF2p immunostaining shows that it could be detected in human colon, prostate, lung, and breast tumors at an early stage (De Luca et al., 2016). Using MMTV-PyVT transgenic mice as a well-defined model of breast cancer, Gualtieri and colleagues found that ORF2p could be detected in the early stage of breast cancer, and its expression level increased with the development of cancer progression (Gualtieri et al., 2013). These two studies indicate that ORF2p might be considered a histological hallmark representing malignancy grades. Moreover, ORF2p, a reverse transcriptase protein, has the potential to trigger heightened SINE retrotransposition when overexpressed (Wallace et al., 2008; Gualtieri et al., 2013). In addition, the reverse-transcription inhibitor efavirenz for anti-HIV therapy inhibits LINE-1 retrotransposition and has been suggested to have anticancer properties in prostate and breast cancer cells (Mangiacasale et al., 2003; Patnala et al., 2014). The EC50 of efavirenz for inhibiting LINE1 reverse transposase activity was approximately 10–5 M (Dai et al., 2011). A novel strategy that combined antiretroviral drugs zidovudine and efavirenz in clinically relevant doses almost entirely blocked tumorigenesis of the colorectal liver metastases mouse model with hyperactivation of LINE-140 (Schneider et al., 2021). Although more studies and clinical trials are needed to test these new findings, these data suggest that ORF2p could not only be considered as a cancer biomarker but also be utilized as a target to develop potential therapies.

The primary regulators that control TE transcription are epigenetic alterations, including both DNA methylation and histone modifications. LINE-1 promoter hypomethylation is a notable hallmark of cancer. In 1993, Thayer and colleagues first revealed that the methylation status of the LINE-1 promotor region is related to LINE-1 activation in cancer cells (Thayer et al., 1993). This finding prompted numerous studies aimed at assessing the connections between LINE-1 hypomethylation and carcinogenesis. In a study involving 203 resected gastric cancer specimens, it was observed that LINE-1 methylation levels are markedly reduced in gastric cancers compared to normal gastric mucosa tissues (Shigaki et al., 2013). It also indicates that hypomethylation in gastric cancer is associated with shorter survival (Shigaki et al., 2013). Two studies in esophageal squamous cell carcinoma show that LINE-1 hypomethylation could trigger carcinogenesis through chromosomal instability and is associated with a shorter survival rate (Iwagami et al., 2013; Kawano et al., 2014). Furthermore, a systematic review and meta-analysis involving 6,107 samples revealed significantly lower LINE-1 methylation levels in tissue samples from cancer patients compared to those from healthy controls. However, this difference was not observed in blood samples (Barchitta et al., 2014).

#### 2.1.2 Timing of transposition and tumor evolution

At what point do somatic LINE-1 insertions start to contribute to tumorigenesis and cancer progression? Human tumors exhibit significant levels of cellular and genetic heterogeneity (Hanahan and Weinberg, 2011). The concept of oncogenic cooperation suggests that it can occur not only within the same cell but also across different cells within the tumor microenvironment (Wu et al., 2010) Suppose 1 cell accumulates some mutation early, which induces a non-autonomous overgrowth of the nearby cell. In that case, it might be hard to figure out the relationship between the mutants and tumorigenesis. A novel APC insertion discovered in 2016 has been described as an early driver of tumor initiation (Scott et al., 2016). Thus, through the “copy-and-paste” mechanism, LINE-1 activity can rapidly increase the copy number and expression level of host oncogenic or tumor suppressor genes, ultimately initiating tumor formation. A study in esophageal adenocarcinoma and its precursor Barrett’s esophagus shows that somatic LINE-1 retrotransposition occurs and aggregates in some histologically normal esophagus cells before esophageal adenocarcinogenesis (Doucet-OHare et al., 2015).

Stress and other harmful environmental stimulations can induce hypomethylation of the human genome and lead to the reactivation of TEs such as LINE-1 (McGowan et al., 2009; Steptoe and Kivimaki, 2013; Kivimaki et al., 2015; Palma-Gudiel et al., 2015; Braun et al., 2016). Analysis of a few highly active LINE-1 loci demonstrates that most retrotransposition events are likely harmless (Tubio et al., 2014). However, as elucidated by the case of the insertion of LINE1 in APC, aberrant L1 retrotransposition events in tumors could be a significant contributing factor to disease progression in some cancer patients (Doucet-OHare et al., 2015). Collectively, these findings indicate that transposable elements, like LINE-1, may play a role in both the initiation and evolution of tumors. Nonetheless, this contention remains a topic of vigorous debate (Lee et al., 2012; Tubio et al., 2014).

#### 2.1.3 DNA transposase is involved in childhood cancers

**FIGURE 3:**
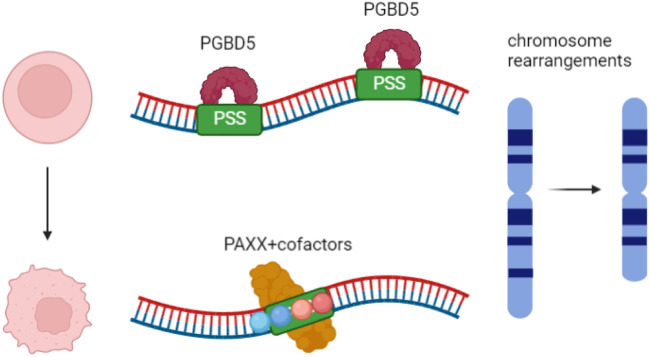
PGBD5 induced chromosome rearrangements and childhood tumor formation. PGBD5 could recognize genomic PSS sequences and induce cell transformation through PAXX-meditated end-joining DNA repair.

DNA transposons, a critical component of the human genome, have been employed in the cellular V(D)J antigen receptor recombination system (Fugmann et al., 2000; Gellert, 2002). In 2013, Majumdar and colleagues found that the human THAP9 gene that encodes an active transposase can mobilize P-Element DNA in human cells (Majumdar et al., 2013). This discovery indicates that some DNA transposase might also have some functions in the human genome. Recently, the human piggyBac transposable element derived 5 (PGBD5) gene was identified as encoding a functional DNA transposase that can catalyze the transposition of synthetic DNA transposons in human cells (Henssen et al., 2015). PGBD5 is primarily, if not exclusively, expressed in the mouse and human brain and central nervous system. It was domesticated over 500 million years ago and is highly conserved from cephalochordates to humans (Pavelitz et al., 2013). Subsequently, PGBD5 was found to exhibit high expression levels in pediatric cancers, notably in rhabdoid and other solid tumors. Overexpression of PGBD5 leads to site-specific DNA rearrangements and is sufficient to induce primary-cell tumor transformation both *in vitro* and *in vivo* (Henssen et al., 2017) (Figure 3). These findings provide a field of view to study the relationship between tumorigenesis, DNA rearrangement, and DNA transpositions.

### 2.2 Involvement of TEs in human genetic diseases

Over 120 TE-mediated germline insertions have been linked to human genetic diseases, with Alu elements accounting for over 60% of these cases and LINE-1 elements contributing to nearly 24% of the total instances (Hancks and Kazazian, 2016). Inherited TE insertional mutations in the germline can cause genetic diseases by disrupting the functions of nearby genes. In 1988, the first TE insertion was identified in the factor VIII gene in two Hemophilia A patients by Kazazian and colleagues (Kazazian et al., 1988). The target site found in exon 14 of the factor VIII gene was inserted by a 3’ terminal of the LINE-1 sequences with its poly A) tract (Kazazian et al., 1988). These findings suggest that LINE-1 can induce disease by utilizing reverse transcription to randomly insert its RNA intermediates, thereby disrupting crucial genes (Kazazian et al., 1988). Since then, many insertional mutations associated with Hemophilia A and Hemophilia B, which are X-linked severe bleeding disorders, have been reported, including mutations in the factor VIII and factor IX genes caused by Alu, SVA, and LINE-1 (Wulff et al., 2000; Li et al., 2001; Sukarova et al., 2001; Ganguly et al., 2003; Mukherjee et al., 2004; Green et al., 2008; Nakamura et al., 2015). Another example of TE insertion at the population level is a homozygous Alu insertion in exon 9 of the male germ cell-associated kinase (MAK) gene as a cause of retinitis pigmentosa in the Jewish population (Tucker et al., 2011). Retinitis pigmentosa (RP) is a common form of inherited retinal dystrophy (IRD) characterized by the apoptotic death of photoreceptor cells. This insertion results in improper splicing of MAK transcript, thereby preventing the formation of mature retinal cells (Tucker et al., 2011).

Most TEs are fixed insertions in the human genome at a high copy number. This abundance of retrotransposon-derived DNA provides ample substrates for disease-producing DNA structural rearrangement and deletion events. Alu recombination is associated with a lot of human genetic diseases. For example, in a patient with Lesch-Nyhan syndrome, an Alu-mediated mutation in hypoxanthine-guanine phosphoribosyltransferase (HPRT) was identified. In this mutation, two Alu elements, respectively, in intron one and intron 3 of paired chromosomes, exchanged from one to the other and formed a non-functional HPRT transcript with duplicated exon two and exon 3 (Brooks et al., 2001). Over extended periods of time, TE-mediated DNA recombination plays a significant role in driving the emergence of new species. For instance, by comparing human and chimpanzee genomes, researchers have identified 492 human-specific deletions mediated by Alu recombination. Thus, TEs-mediated DNA recombination should not be considered as isolated or selective events but rather as cumulative contributions to changes in the human genome (Sen et al., 2006).

### 2.3 Involvement of TEs in aging and immune reaction

Ging is a biological process that leads to a decline in vitality and health over time by accumulating multiple different molecular and cellular damages. It increases the risk of numerous disorders, including Alzheimer’s disease, diabetes, hypertension, cardiovascular disease, cancer and many more. A defining feature of aging is the dysfunction of fundamental biological structures, including chromatin (Oberdoerffer and Sinclair, 2007; Lopez-Otin et al., 2013). Notably, in *Drosophila*, two repressive markers, H3K9me3 and HP1, have been observed to decrease as individuals age, particularly in heterochromatic regions (Wood et al., 2010). The age-related reduction in silencing within heterochromatic regions can lead to increased activation of TEs since TEs are predominantly concentrated and suppressed in these areas (Chen H. et al., 2016). The disruption of TE silencing in somatic cells can result in the accumulation of TE DNA sequences, thereby triggering innate immune responses. This hyperactivation of the immune system can expedite the aging process. Moreover, the loss of TE silencing in somatic cells can also promote somatic mutagenesis, potentially leading to age-related disorders and diseases.

In mammals, the type I interferon (IFN) response serves as the primary line of defense against invading viruses, activated upon detecting the presence of these viral invaders. Hence, mammalian cells have established a series of sensors that could recognize typical characteristics of viral nucleic acids in the cytoplasm and trigger the activation of the type I IFN response. However, due to the similarities between TE-derived and viral nucleic acids, cells can misidentify TEs-triggered hyperactivation as viral infection and induce the same type I IFN response. Recently, a study analyzing the Gene Expression Omnibus database has described an interesting phenomenon that genome-wide transposon upregulation is associated with virus infections in humans and mice (Macchietto et al., 2020). It indicates that viral infection and TE activation share similar signal pathways.

**FIGURE 4:**
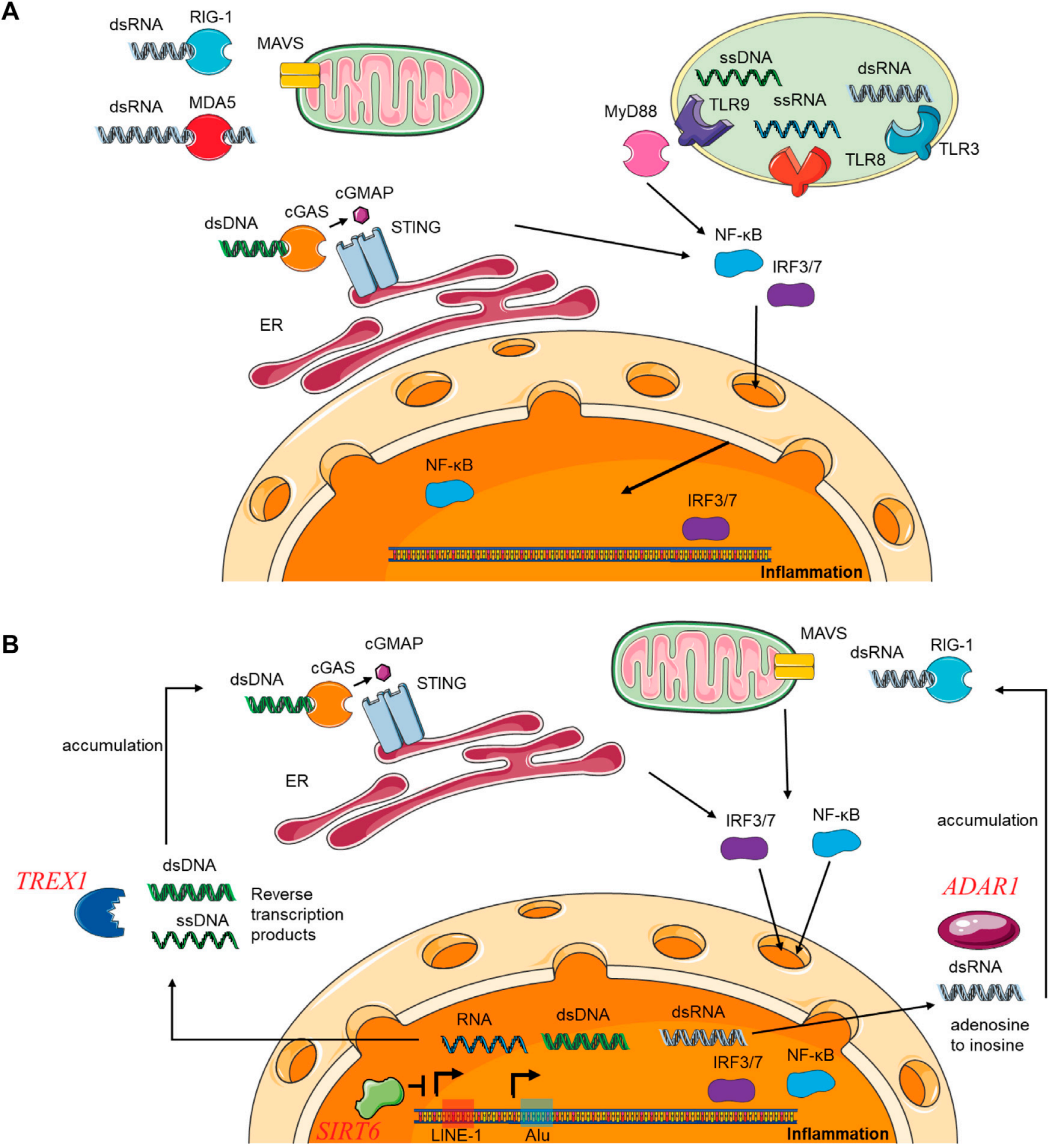
The cytosolic DNA and RNA mediated cGAS-STING pathway in innate immunity under conditions of infection and TE accumulation. **(A)** The mechanisms of free DNA and RNA sensing exist in mammalian cells and induce inflammation through the cGAS-STING pathway. **(B)** TEs DNA sequences and RNA intermediates could accumulate in the cytoplasm under conditions in Aicardi–Goutières syndrome, and induce cGAS-STING pathway and inflammation. Abbreviations: dsDNA, double-stranded DNA; dsRNA, double-stranded RNA; ssDNA, single-stranded DNA; ssRNA, single-stranded RNA; cGAS, Cyclic GMP-AMP synthase; STING, Stimulator Of Interferon Response CGAMP Interactor.

Here, we will first describe the mechanisms of free DNA and RNA sensing existing in mammalian cells (Figure 4A). Sensors to detect viral genomes or TE-derived intermediates are widely expressed in the cytoplasm and restricted to specific cell types as receptors in the cell surface and endosomal compartments. The cyclic GMP-AMP synthase (cGAS) is the primary pathway to detect cytoplasmic viral double-strain DNA (dsDNA). It triggers the formation of cyclic guanosine monophosphate–adenosine monophosphate (cGAMP), which activates the adaptor protein STING, promotes the nuclear translocation of interferon regulatory factor 3 (IRF3), IRF7, and nuclear factor κB (NF-κB), and induce interferons expression (Sun et al., 2013; Wu et al., 2013). Moreover, single-strain DNA (ssDNA) can also induce a weak activation of the cGAS-STING signal pathway (Chen Q. et al., 2016). A Toll-like receptor, TLR9, mediates the recognition of unmethylated ssDNA and promotes the secretion of type I IFNs and proinflammatory cytokines through similar downstream signal pathways (Hemmi et al., 2000; Kawai et al., 2004; Hoshino et al., 2006). The cytoplasmic retinoic acid-inducible gene I (RIG-I)-like receptors (RLR), which includes RIG-I, melanoma differentiation-associated gene 5 (MDA5), and laboratory of genetics and physiology 2 (LGP2), are vital sensors to detect viral double-strain RNA (dsRNA) (Cuellar et al., 2017). RIG-I and MDA5 favor detecting short dsRNAs and long dsRNAs, respectively. Both subsequently engage with the mitochondrial antiviral signaling (MAVS) protein, initiating the nuclear translocation of IRF3, IRF7, and NF-kB, thereby stimulating the expression of IFNs and cytokines (Hornung et al., 2006; Pichlmair et al., 2006; Kato et al., 2008; Myong et al., 2009; Schmidt et al., 2009; Feng et al., 2012; Goubau et al., 2014). TLR3 can also medicate the recognization of dsRNA and trigger the same IFNs and cytokines pathways (Alexopoulou et al., 2001). Another toll-like receptor, TLR8, can bind and recognize ssRNA and induce type I IFN response with a similar signal pathway, but a myeloid differentiation primary response 88 (MyD88) is needed (Diebold et al., 2004; Heil et al., 2004; Greulich et al., 2019; Ostendorf et al., 2020).

TEs DNA sequences and RNA intermediates could accumulate in the cytoplasm under conditions such as Aicardi–Goutières syndrome (AGS) (Figure 4B). AGS is a heritable form of autoimmune disease, and many loss-of-function mutations in TREX1, SAMHD1, RNASEH2A, and ADAR1 genes can cause it. Each of these four genes, associated with distinct stages of nucleic acid metabolism, has been documented to hinder the transposition of LINE-1 (Stetson et al., 2008; Zhao et al., 2013; Orecchini et al., 2017; Benitez-Guijarro et al., 2018). In addition, ADAR1 catalyzes the deamination of dsRNA molecules, and its loss of function in AGS would induce the accumulations of not only LINE-1 dsRNA intermediates but also Alu dsRNA intermediates and increase IFN activation through MDA5/MAVS signaling pathway (Wang et al., 2000; Mannion et al., 2014; Liddicoat et al., 2015; Pestal et al., 2015; Benitez-Guijarro et al., 2018). In humans, TREX1-deficient neural cells, neurons, and organoids represent the abundant extrachromosomal accumulation of LINE-1, SINE, and other TEs cDNAs (Thomas et al., 2017).

During cellular senescence in human fibroblasts, LINE-1 transcription becomes activated, leading to the accumulation of LINE-1 intermediates. This activation has been attributed to the involvement of three factors: TREX1, RB1, and FOXA1 (De Cecco et al., 2019). In SIRT6-deficient mice, abundant cytoplasmic LINE-1 cDNA is accumulated in all cells and tissues, which also triggers strong type I interferon response and short lifespan (Simon et al., 2019). In each of the conditions above, accumulation of LINE-1 dsDNAs and ssDNAs are detected by cGAS, subsequently prompting the activation of type I IFN responses. Notably, treating mice with inhibitors of the LINE-1 reverses transcriptase, and knocking down LINE-1 expression downregulates IFN activation and declines disease or age-related inflammations. These findings elucidate the connections between TE activation, inflammation, autoimmune diseases, and aging.

## 3 TEs as a tool for biological application

TEs have been utilized as powerful genetic tools to reliably and effectively introduce an array of foreign DNA sequences into genomes. This includes selectable markers, epitope/fluorescent reporters, barcode cassettes, mutagenic gene or enhancer trap cassettes, shRNA expression cassettes, and therapeutic constructs. Three DNA transposon systems, namely, Tol2, Sleeping Beauty (SB), and piggyBac (PB), stand as the most extensively employed techniques for genome manipulations in vertebrates. These systems have been used for genome manipulations in vertebrates, such as integrating foreign genes into tissue culture cells, generating transgenic animals, conducting forward genetic screens for functional gene annotation, and serving as therapeutic carriers to introduce beneficial genes into individuals with genetic disorders. Other novel transposons discovered in fish also exhibit similar behaviors as a genetic editing tool (Shen et al., 2021; Wang et al., 2023). SB has high activity in all vertebrates, but Tol2 only presents high activity in zebrafish germline cells (Kawakami et al., 2004; Parinov et al., 2004; Rafferty and Quinn, 2018). PB has high activity in yeast, plants, and humans and is a valuable tool for mutagenesis throughout species (Handler, 2002; Gonzalez-Estevez et al., 2003; Lorenzen et al., 2003; Sarkar et al., 2003; Sumitani et al., 2003; Thibault et al., 2004; Ding et al., 2005; Chang et al., 2019; Levitan et al., 2020). Here, we will focus on the biological application of SB and PB in mammals. The remobilization mechanism of DNA transposons such as SB and PB could be described as “cut-and-paste.” More precisely, a transposase enzyme recognizes and binds to the inverted repeat sequences (ITRs) in the transposon and mediates transposition by initiating excision and the following reintegration into a new locus. SB, a synthetic Tc1-like transposon, has been “woken up” by removing the inactivating mutations of a transposase gene from the salmonid subfamily in 1997 (Ivics et al., 1997). Following its reconstruction, it has been proven to mediate transposition and insertion at TA target sites in mouse and human cells (Ivics et al., 1997; Luo et al., 1998; Izsvak et al., 2000). PB was originally discovered in the cabbage looper moth Trichoplusia ni123. It is a 2,472 bp transposon featuring two 13 bp ITRs and a 594 amino acid sequence. Notably, PB prefers insertion at TTAA target sites (Cary et al., 1989; Fraser et al., 1995; Fraser et al., 1996). In the following, we will focus on two major applications of TEs, including functional screening in cancer and phenotypic screening in the whole genome.

With the development of high-throughput sequencing technology, the Tn5-based transposon system has been innovatively applied to improve the efficiency of sample processing for high-throughput sequencing. For example, an assay for transposase-accessible chromatin using sequencing (ATAC-seq) has been developed to utilize the Tn5 transposon system to detect open chromatin sites in mammalian cells (Buenrostro et al., 2013). Linear amplification via transposon insertion (LIANTI) uses a Tn5 transposon system for single-cell genomic analyses (Chen et al., 2017). Here, we will review the recent technological innovations combining transposon-based amplification and high throughput sequencing.

The Clustered Regularly Interspaced Palindromic Repeats (CRISPR-Cas) system, an adaptive immunity system found in most archaea and many bacteria, is currently the most suitable strategy for genome editing in mammalian cells (Jinek et al., 2012; Cong et al., 2013; Mali et al., 2013). Recent bioinformatics analysis has revealed a superfamily of CRISPR–Cas encoded DNA transposons, in which transposons are predicted to spread in an RNA-guided manner (Peters et al., 2017; Faure et al., 2019). The emerging CRISPR transposon system is expected to be a valuable asset for the site-specific integration of DNA sequences into mammalian genomes. At the same time, a gene delivery tool (Find and cut-and-transfer (FiCAT)) has been developed to combine CRISPR-Cas9 system and PB transposon system for precise targeted insertions in mammalian genomes with high efficiency (∼20%) (Pallares-Masmitja et al., 2021). Here we will summarize the status of this new field and try to figure out the possible directions in the future.

### 3.1 TEs-based *in vivo* screen for functional cancer genomics

**FIGURE 5:**
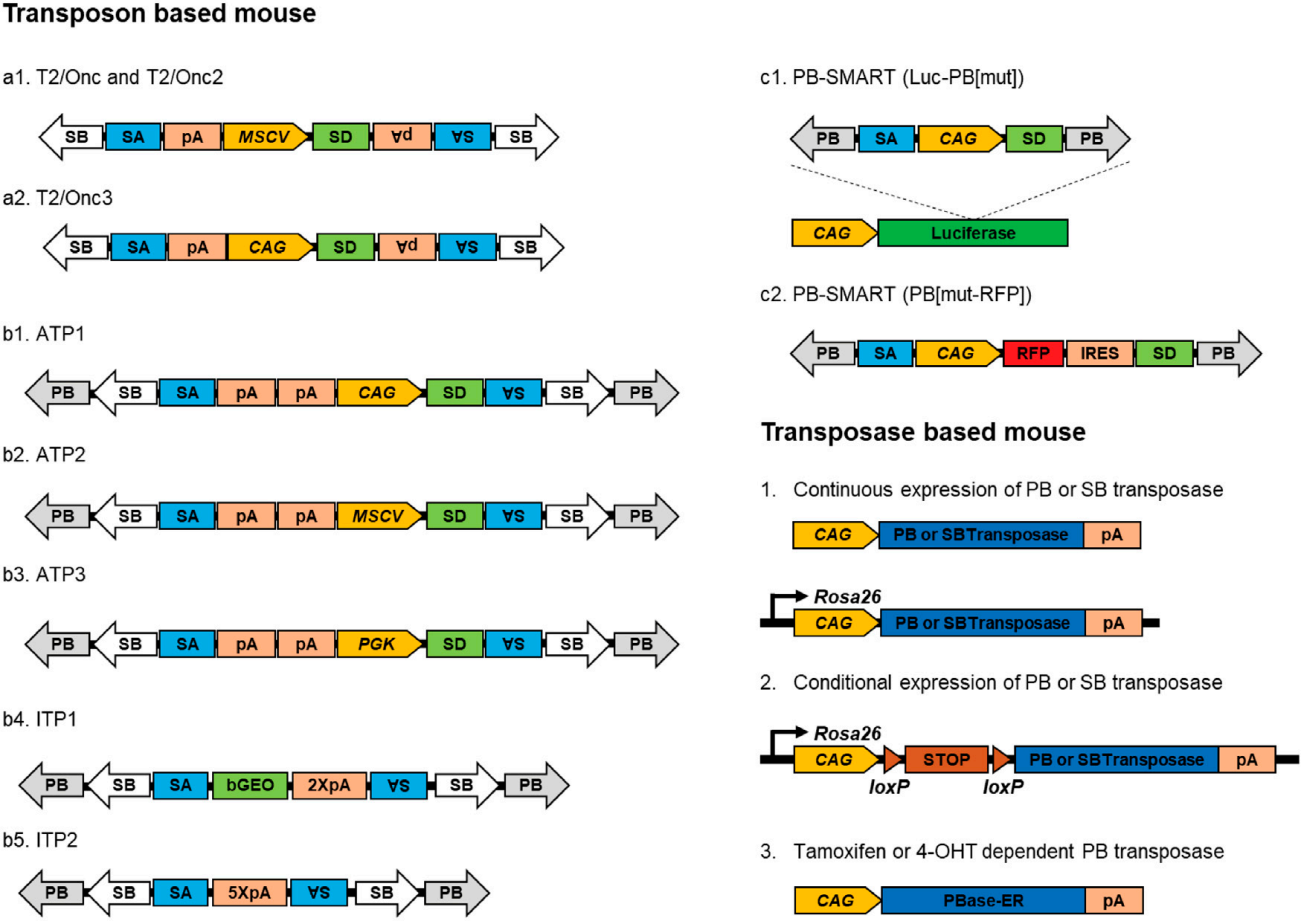
Transposon tools for cancer genomic screening in mice. Transposon and transposase-based mouse lines for cancer genomic screening; SB-based transposon mouse lines include T2/Onc, T2/Onc2, and T2/Onc3 (a1, a2); SB and PB-based transposon mouse lines include ATP1, ATP2, ATP3, ITP1, and IT2 (b1-b5); PB based transposon mouse lines PB-SMARTs (c1 and c2).

In 2005, two pioneering studies demonstrated that somatic mutagenesis induced by engineered SB transposition platforms in mice could be applied to discover novel cancer genes (Collier et al., 2005; Dupuy et al., 2005). SB-driven somatic insertion sites in solid tumors can be easily cloned and quickly analyzed, enabling the identification of novel genes involved in tumor development through a forward genetic approach. The platform consisted of two transgenic mouse strains: the ‘transposase mice’ and ‘transposon mice’. In transposase mice, SB transposase is expressed under the control of either the CAGGS promoter or through insertion into the Rosa26 locus. In transposon mice, both of the two mutagenic transposon vectors have been designed using similar components, including splicing acceptors followed by a polyadenylation signal in both orientations for disrupting transcription and a strong promoter from the murine stem cell virus (MSCV), with a splicing donor in the middle of the construct for overexpressing downstream genes. After crossing these two mouse strains, the double-transgenic mice (T2/Onc2/Rosa26-SB11) exhibited higher embryonic lethality and smaller size. By 17 weeks, all double transgenic mice died from cancer with various tumor types, including T-cell, B-cell lymphoma, medulloblastoma, intestinal and pituitary neoplasia, and others. The other double transgenic mice (T2/Onc/CAGGS-SB10) do not exhibit cancer susceptibility but do exhibit accelerated tumorigenesis when carried on the tumor suppressor p19Arf deficient background. The difference in cancer susceptibility between T2/Onc2/Rosa26-SB11 and T2/Onc/CAGGS-SB10 might be caused by either of the following: a) T2/Onc2 mice carry almost 10-fold higher copy number of mutagenic transposon vectors than T2/Onc; b) Rosa26-SB11mice express transposase in a higher level than CAGGS-SB10 (Figure 5).

In 2010, Rad and colleagues reported a new design of mutagenic transposon vectors that combines both PB and SB transposition systems to maximize the utility of their different integration preferences (Rad et al., 2010). In transposon mice, three mutagenic transposons (ATP1, ATP2, and ATP3) are designed with different promoter/enhancer elements (CAG, MSCV, and PGK), which permit gain-of-function or loss-of-function mutations (Figure 5). Following the crossbreeding with RosaPB mice, RosaPB; ATP1 mice bearing the CAG promoter succumbed to a high incidence of solid tumors, encompassing sarcomas and diverse carcinomas. By contrast, more than 90% of cancers developed by RosaPB; ATP2 mice are aggressive leukemias and lymphomas (Rad et al., 2010). Later in 2011, Sean and colleagues developed a PB-only transposition system for somatic mutagenesis with an activated reporter and tracker (PB-SMART) (Figure 5). PB-SMART mice can label over-proliferative cells with somatic mutations by bioluminescence or red fluorescence, which facilitates tracking the location, growth, and infiltrations of tumor cells (Landrette et al., 2011).

Constitutive expression of PB or SB transposase triggers whole-body mutagenesis, which causes a broad spectrum of tumor types. A high percentage of blood cancers have been induced by the mutagenic transposon carrying the MSCV promoter, which has intense activity in the early stage of the hematopoietic lineage (Dupuy et al., 2005; Rad et al., 2010). Re-engineering of mutagenic transposon variants with different promoters biases oncogenesis toward forming solid cancers in double transgenic mice (Dupuy et al., 2009; Rad et al., 2010). To induce insertional mutagenesis in specific tissues, organs, or cell types of interest, conditional SB or PB transposition systems have been engineered. These systems facilitate tissue-specific screens and have led to numerous groundbreaking discoveries (Dupuy et al., 2009; Starr et al., 2009; Vassiliou et al., 2011). As an example, in order to induce somatic mutagenesis in the pancreas, a novel mouse strain was created. In this strain, the PB transposase DNA was integrated into the *Rosa26* locus, with a *lox*P-flanked stop cassette (LSL) positioned in between. This study successfully unveiled a set of driver genes specific to pancreatic cancer, proving challenging to identify using alternative approaches (Rad et al., 2015).

The interaction between genetic and environmental factors plays a pivotal role in both tumorigenesis and the progression of cancer. Transposon-based mutagenesis provides an efficient way to dissect the involvements of different genes in different contexts. For example, a series of SB transposon mutagenesis screens have been performed in the intestine of mice with different oncogenic mutations (APC (Apcmin), KRAS (KrasG12D), SMAD4 (Smad4KO), and TP53 (p53R172H)) that mimic different stages of colorectal cancer (CRC) developments. This approach allowed us to understand better the evolutionary forces driving different stages of CRC progression (Takeda et al., 2015). Other SB or PB-based mutagenesis screens have been performed with various oncogenic conditions, including a constitutive knock-out *Arf* model (Collier et al., 2005), a conditional knock-in ‘humanized’ *NPM1c* model (Vassiliou et al., 2011), a conditional knock-in *Braf* ^*V600E*^ model (Ni et al., 2013), a conditional knock-out *Cdh1* model (Kas et al., 2017) in both whole body and tissue-specific tumors that discovered a lot of new therapeutic gene target candidates. Hepatitis B virus (HBV) infection in humans is the fastest-rising cause of hepatocellular carcinoma (HCC) developments. Another study has achieved a near-saturating SB mutagenesis screening in a chronic HBV-HCC model and has identified 21 early-stage candidate drivers and 2,860 late-stage candidate drivers which provides a comprehensive outline of the genetic landscape of HCC in an HBV-infected and chronic inflammation context (Bard-Chapeau et al., 2014). A series of SB screens for HCC have been performed in a variety of other conditions including nonalcoholic fatty liver disease (PTEN conditional knock-out model), hepatic steatosis-inducing diet, and chronic liver injury (CCl_4_ model) that identified distinct environmental signal pathways involved in HCC tumorigenesis (Tschida et al., 2017; Kodama et al., 2018; Riordan et al., 2018).

Mutagenesis screens based on the SB have been instrumental in unraveling the mechanisms underlying cancer recurrence. Cancer recurrence refers to a situation in which a patient, previously believed to be cancer-free following surgical removal and drug treatment, experiences the return of cancer. For instance, a study using an SB transposon-driven medulloblastoma mouse model (*Ptch* ^+/−^/*Math1-SB11*/*T2Onc* or *T2Onc2*) with micro-neurosurgical tumor resection and image-guided radiotherapy identified a very poor overlap between primary tumors and their recurrences (Morrissy et al., 2016). This genetic divergence of the dominant clones before and after treatment was also confirmed in human medulloblastoma samples (Morrissy et al., 2016). SB-based mutagenesis screens have also helped to discover drug resistance genes. A study using SB insertional mutagenesis in mice conditionally expressing Braf (V618E) identifies drivers of melanoma formation and mediators of resistance to the BRAF inhibitor plx4720, including *Braf, Mitf*, and *ERas* (ES-cell expressed Ras) (Perna et al., 2015). Given the rapid increase of new targeted therapies for different genotypes of cancers, a critical issue will be to overcome treatment relapse. Therefore, the requirement for experimental systems capable of deciphering cancer recurrence and drug resistance mediators will continue to expand.

### 3.2 TE-based phenotype-driven screen

TEs have been used as phenotype-driven screening tools in invertebrates, including *Yeast*, *Caenorhabditis elegans,* and *Drosophila melanogaster* for many years, which help to discover critical genes and pathways in various biological circumstances (Rubin and Spradling, 1982; Handler et al., 1993; Spradling et al., 1995; Ross-Macdonald et al., 1999; Kumar et al., 2002a; Kumar et al., 2002b; Jorgensen and Mango, 2002; St Johnston, 2002; Castano et al., 2003; Uhl et al., 2003; Thibault et al., 2004). Compared with traditional chemical treatments for DNA mutagenesis, TE-based insertional mutagenesis is an attractive alternative approach for genome-wide phenotypic screens in mammals, which provide a marked and regulated mutation that can be easily recognized by mapping the sequences of transposon itself. Using the mouse model as an illustration, mouse mutants can be created by manipulating embryonic stem (ES) cells. This manipulation involves knocking out specific genes through homologous recombination and employing the RNA-guided CRISPR/Cas9 knockout technique. However, both approaches remain too expensive to generate a genome-wide mouse mutant library for phenotypic screening. In contrast, TEs can be used to produce a large or even saturating number of mutations in multicellular organisms faster and at a lower cost, although “local hopping” behavior and transposition preference can influence data analysis.

**FIGURE 6:**
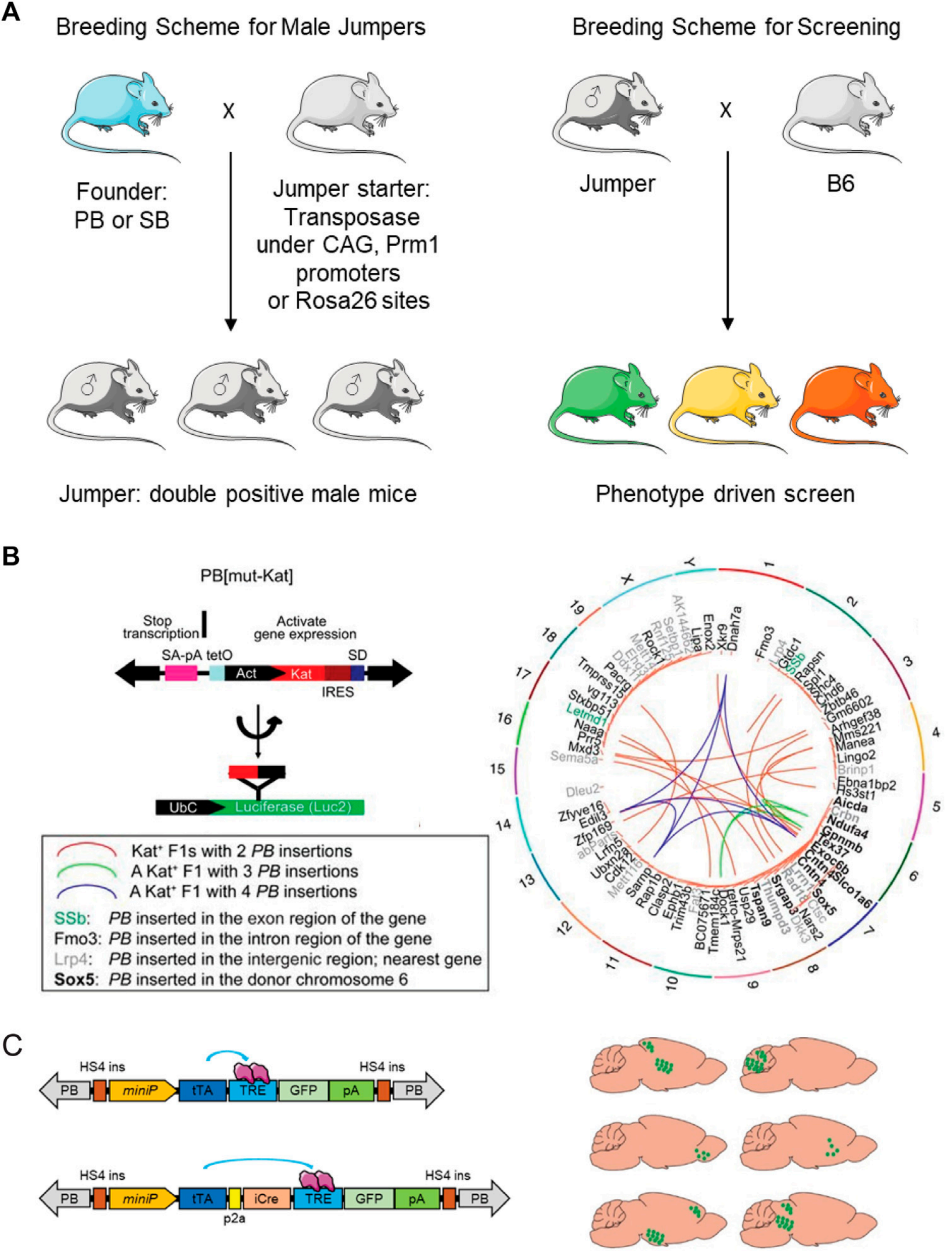
Transposon tools for phenotype-driven screening in mice. **(A)** Breeding scheme of phenotype-driven screening; **(B)** Transposon design and mutagenesis ability for phenotype drive screening; **(C)** Transposon design for brain region randomly labeling.

SB was first tested in mice for insertional mutagenesis (Dupuy et al., 2001; Fischer et al., 2001; Horie et al., 2001; Horie et al., 2003). The breeding scheme is described above in sections summarizing (Fischer et al., 2001; Horie et al., 2001) (Figure 6A). First, two independent transgenic mice were established, including a founder integrated with a single copy or multiple copies insertion of SB transposon and a jump-starter integrated with SB transposase under the control of Actin promoter (whole body) or Prm1 promoter (germline-specific). Second, the jumper mouse was bred from the cross of the founder mouse with the jump-starter mouse. In the germline of the double-positive jumper mouse, SB transposase would help SB transposon to remobilize into a new site of the genome. Following the mating of the jumper with *wild-type* female mice, a population of transgenic mice carrying SB insertions at new sites will be generated.

The SB system was first employed in a region-specific saturation insertional mutagenesis screen in mice (Keng et al., 2005). In this paper, Vincent and colleagues first demonstrated that the transposition local hopping is evident in the SB system, with ∼63% transposition events happening on the donor-site chromosome. This phenomenon provides strong support for a saturated screen in donor chromosomes, comparable to the mutagenesis efficiency of ENU-mediated mutagenesis. Nine of 31 mutant lines characterized by homozygosity have been detected with phenotypes including embryonic lethal, postnatal lethal, or postnatal nonlethal.

In 2011, Ruf and colleagues introduced an SB-based transposition system that offers an efficient method for systematic *in vivo* analysis of regulatory elements within mammalian chromosomes (Ruf et al., 2011). This system uses an SB transposon containing a LacZ reporter and a mini-promoter from the human *β*-globin gene with the help of SB transposase to integrate into the genome randomly. By performing LacZ staining on E11.5 embryos, the authors found that many embryos exhibited tissue-specific expression of the reporter gene (Ruf et al., 2011). This new attempt provides a genetic tool with many possibilities, including inducing large fragment deletion combined with a recombination system and producing a large number of Cre or Flpo-dependent tissue-specific lines randomly.

Compared to the SB system, the PB system exhibits higher accuracy of excising and re-integrating and less “local hopping”. Leveraging the advantages of the PB system, a large-scale mutagenesis screen in mice generated more than 5,000 mutant mouse strains (Sun et al., 2008; Cui et al., 2016). This method was employed to conduct an initial genetic screen for obesity-related mutations, leading to the discovery of an orphan G protein-coupled receptor (GPCR), GPR45. This receptor was found to be associated with obesity and hepatic steatosis through its regulation of pro-opiomelanocortin (POMC) expression (Cui et al., 2016). Later, Chang and colleagues generated a PB-based first-generation (F1) dominant screening system in mice (Chang et al., 2019). This new approach provides the opportunity to conduct a highly efficient and affordable genome-wide phenotypic screen in a single laboratory. This study presents a new PB construct design with a conditionally regulated strong promoter for gene overexpression and two splicing acceptors for gene disruption. The construct also incorporates a red fluorescent protein to visualize and track the mutant and an interrupted luciferase to monitor the transposition efficiency. The authors further note that if the founder carries ten copies of the PB transposon, the jump-starter could generate genome-wide mutations in 55.2% of F1 progeny, about 4 to 8-fold higher than previous reports (Figure 6B). By this forward genetic mutagenesis screening system, five growth retardation-related genes have been identified, including *Rin2*, *Rbm39*, *Mll*, *Zeb2*, and *Six1/4* in mice (Chang et al., 2019).

The PB-based transposition system has also been used to dissect the neuronal gene network in the mouse brain, which is considered the most complex biological structure, composed of thousands of distinct neuronal cell types (Figure 6C). The idea is to use an “enhancer trap” probe with a minimal promoter and a reporter gene to distribute into the mouse genome randomly. If there exists a specific neuronal cell type associated regulator element nearby, the mini promoter will trigger the expression of the reporter gene to highlight the subgroup of neurons. The critical point of the enhancer trap is to find a suitable mini promoter with a lower expression background and higher sensitivity to nearby enhancers. A thoughtful design that combined the minimal promoter hsp and tet-off operator system has been used to generate Cre-dependent mice through a PB-based transposition system (Shima et al., 2016). This tet-enhancer probe helps to generate a series of mouse lines that have highly restricted expression patterns in the mouse brain and labels many novel neuronal cell types with high efficiency.

Another notable application of the PB-based screening system involved the systematic elucidation of essential genes within the human malaria parasite, *Plasmodium falciparum* (Zhang et al., 2018). Malaria is an acute febrile illness caused by eukaryotic *Plasmodium spp* parasites that disrupt human red blood cells. This study achieves saturation mutagenesis by generating more than 38,000 P. falciparum mutants based on PB and subsequently highlights 2,680 essential genes that would be valuable for antimalarial drug research (Zhang et al., 2018).

### 3.3 Involvement of TEs in high-throughput sequencing

Tn5 transposition system is the most widely used *in vitro* transposition system. In recent years, the Tn5 system has been innovatively applied to high-throughput sequencing. A hyperactivate derivative of the Tn5 transposase can be used to catalyze the transposition of synthetic oligonucleotides into target DNA and to fragment it into pieces with high efficiency (Davies et al., 2000). Random integration and fragmentation make subsequent read assembly more informative and reliable. Here, we will summarize the mechanism and several essential applications of Tn5 transposon.

#### 3.3.1 The mechanism of Tn5 transposition

Tn5 transposon was first discovered in *Escherichia* cdi. Its transposase catalyzes a multi-step “cut and paste” transposition reaction. First, Tn5 transposase binds to specific 19-bp terminal DNA sequences. Next, Tn5 transposase and DNA oligomerize to form a catalytically active synaptic complex. The entire catalytic process comprises two reactions. In the first reaction, the Tn5 transposase utilizes an activated water molecule to initiate a nucleophilic attack, hydrolyzing a 3′end of the transposon. As a result of this step, a 3′OH group becomes exposed at the transposon’s end. Subsequently, this activated 3′OH terminus carries out a nucleophilic attack on the 5′DNA strand, forming a hairpin structure and excising the transposon from the donor DNA. Following that, the hairpin is hydrolyzed, resulting in blunt-ended DNA at the transposon’s end. In the last step, the synaptic complex attaches to the target DNA, and the activated 3′OH groups at the transposon ends initiate a nucleophilic attack on both strands of the target DNA. This final strand transfer event generates a 9-bp sequence duplication immediately flanking the transposon insertion site (Davies et al., 2000).

**FIGURE 7:**
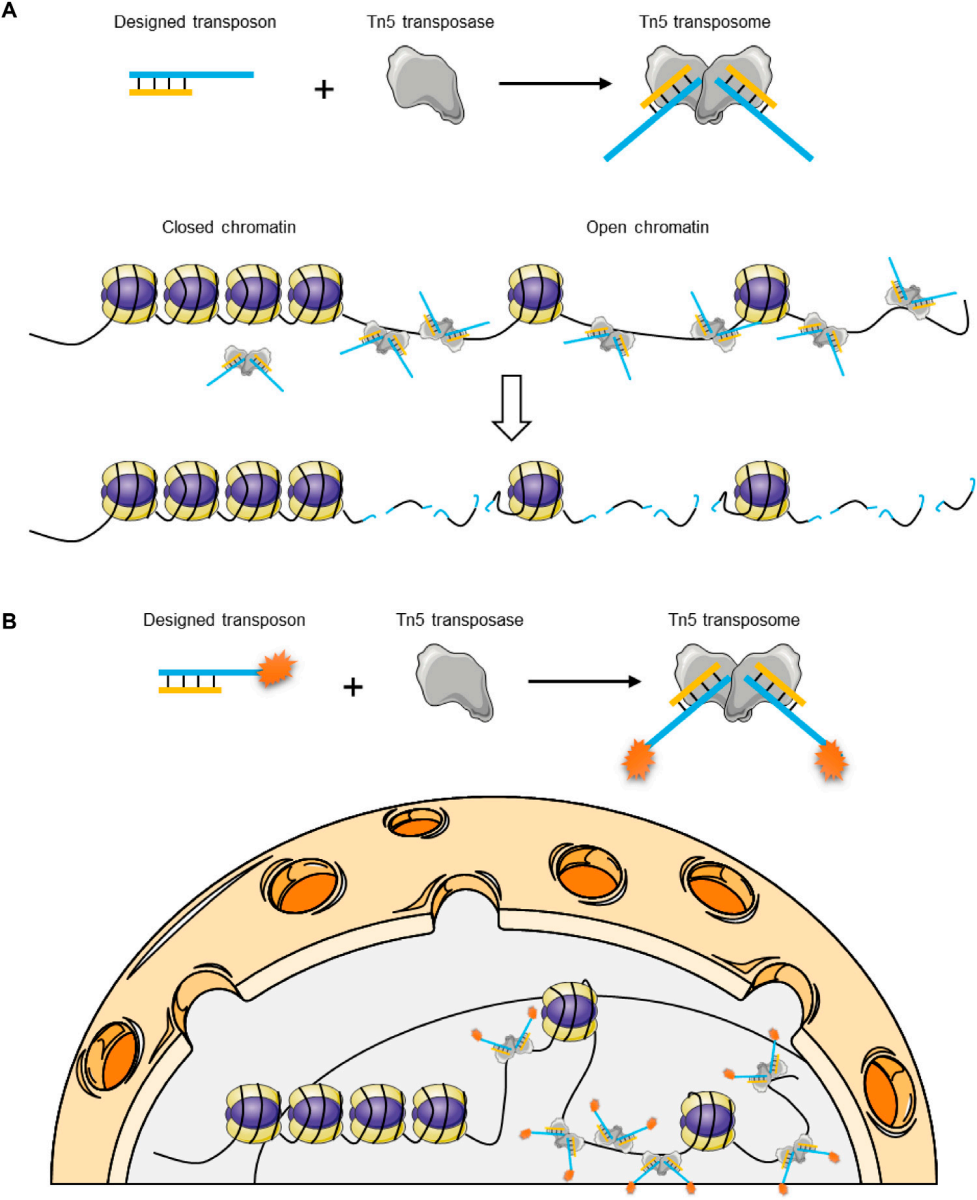
ATAC-seq and ATAC-see overviews. **(A)** Tn5 prefers to bind and cut open chromatin and simultaneously ligates with adapters in the truncated DNA during an ATAC-seq; **(B)** Schematic of ATAC-see. An optimized bifunctional Tn5 transposon with fluorescent adaptors could be used to label open chromatin regions in fixed cells.

Assay for Transposase-Accessible Chromatin using sequencing (ATAC-seq) is a technique to identify regions of open chromatin in the genome, which is based on Tn5 transposition ability *in vitro* (Buenrostro et al., 2013) (Figure 7A). Compared with other methods such as DNase-seq (Song and Crawford, 2010) and FAIRE-seq (Waki et al., 2011), ATAC-seq can offer more accurate, more time-saving, and more sensitive measurements for assaying chromatin accessibility with fewer input cells, and more simple steps (Buenrostro et al., 2013). With the development of the single-cell technique, Greenleaf and colleagues developed a single-cell assay for transposase-accessible chromatin (scATAC-seq) based on the Tn5 transposition system and a programmable microfluidics platform (Fluidigm), enabling the interrogation of the epigenomic landscape of small biological samples from normal tissues or disease models (Buenostro et al., 2015). Barnett and colleagues have developed the ATAC-me technology, which detects accessibility and methylation from the same single preparation (Barnett et al., 2020). Barnett and colleagues have developed the ATAC-me technology, which detects accessibility and methylation from the same single preparation176. For library construction, it first uses Tn5-based methodology to cleave and tag the genome DNA with asymmetric, methylated adapters. Then, a bisulfite treatment of the assembled sample labels methylation sites. To unveil the spatial organization of the genome, many visualization techniques have been developed based on ATAC-seq and Tn5 tagmentation ability, such as ATAC-see (Chen X. et al., 2016), and 3D ATAC-PALM (Xie et al., 2020) (Figure 7B). ATAC-see uses Tn5 transposase coupled to Atto dye-conjugated DNA probes to label open chromatin and uses a confocal microscope to visualize the distributions. In the same way, 3D ATAC-PALM uses Tn5 transposase coupled to bright photoactivatable Janelia Fluor 549 (PA-JF_549_) conjugated DNA probes and PALM super-resolution imaging to visualize the marked open chromatin.

**FIGURE 8:**
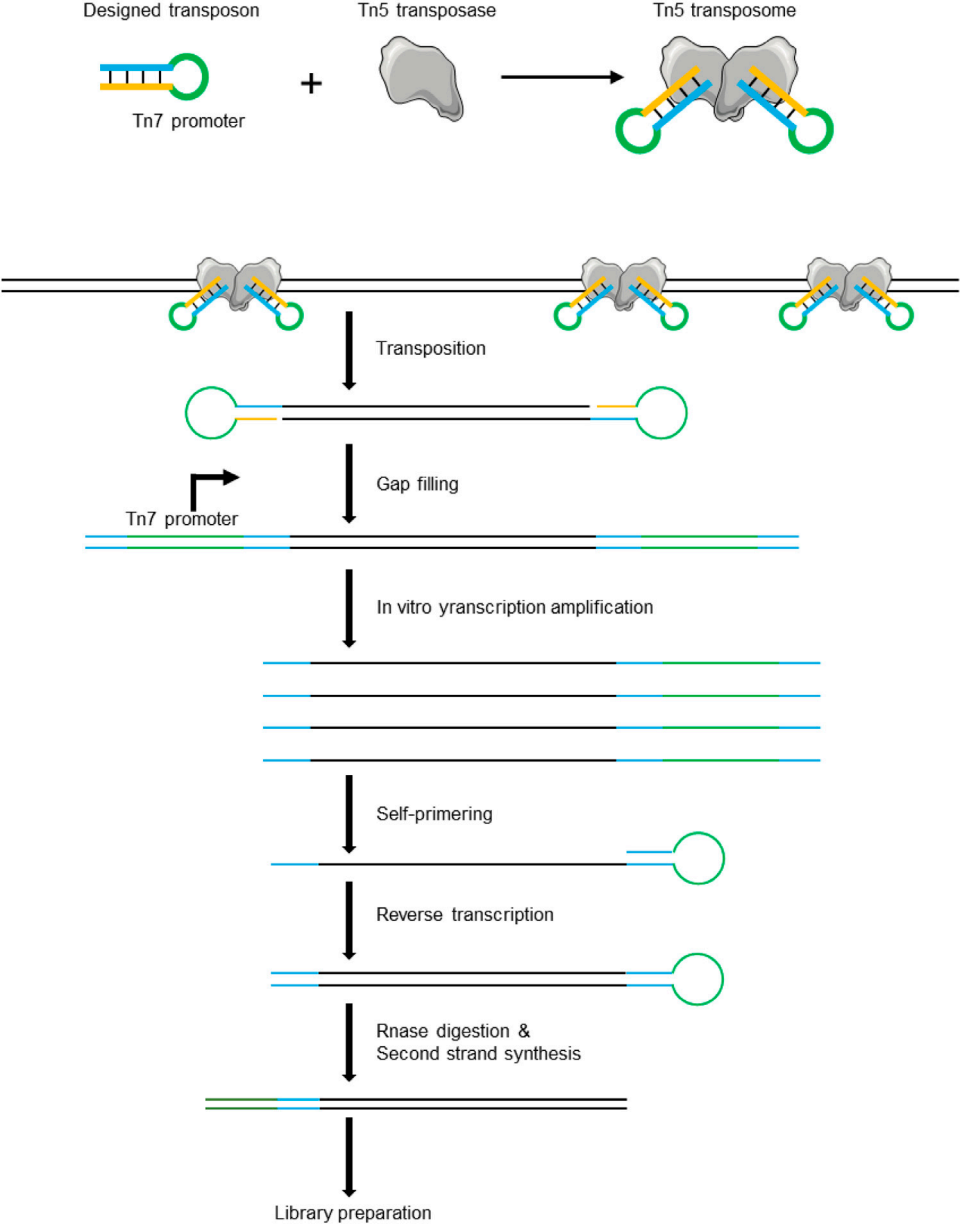
Schematic of LIANTI technology. LIANTI utilizes Tn5 transposase coupled to synthetic hairpin DNAs with T7 promoter to randomly dig the genome of a single cell into pieces.

The critical point for single-cell genome sequencing is how to achieve a whole-genome amplification linearly. LIANTI technology first solved this problem (Figure 8). It utilizes Tn5 transposase coupled to hairpin DNA with T7 promoter to insert into the genome of a single cell (Chen et al., 2017). t utilizes Tn5 transposase coupled to hairpin DNA with T7 promoter to insert into the genome of a single cell128. Tn5 transposase-DNA complex helps to generate a library with tagged DNA pieces. Then, it employs T7 polymerase to obtain a large number of linearly amplified transcripts. This new technique helps to identify single-nucleotide variations (SNVs) in kindred cells. In 2021, Xing and colleagues reported a new single-cell WGA method termed multiplexed end-tagging amplification of complementary strands (META-CS) (Xing et al., 2021). It linearly amplifies both of the strains using the Tn5 transposition system and uses the sequencing data from the complementary strain as a reference to correct the single-cell genome sequencing data. It has achieved the highest accuracy thus far.

### 3.4 RNA-directed transposition

CRISPR-Cas9 system has been widely used for specific target sequences based on the guide RNA. If we can develop a system that could achieve RNA-guided site-specific transposition *in vivo*, it would provide an efficient tool to deliver small and large payloads into the genome.

#### 3.4.1 RNA-guided transposition system developed by protein engineering

In 2019, Hew and colleagues reported the first evidence that PB transposase and dead Cas9 (dCas9) fusion protein could be used to achieve RNA-guided transposition in human cells (Hew et al., 2019). In this paper, the authors attempt multiple designs, including different dCas9 mutants fused with the hyperactive PB transposase and its variants, and they find that dCas9-PB fusion constructs with eight unique gRNAs designed for the human CCR5 safe harbor sequence could help to target a single sequence in the CCR5 gene (Hew et al., 2019). As a proof-of-concept, it establishes an opportunity for improved targeting vectors with potential applications in gene therapy. Meanwhile, Kovač and colleagues described an RNA-guided dCas9 and SB transposase system for targeting transposition (Kovac et al., 2020). In this article, the authors explored three distinct design approaches, which included: 1) dCas9 fused to the N terminal of SB transposase (SB100X); 2) dCas9 fused to the N-terminal of an adaptor domain N57 + SB100X; 3) dCas9 fused to the C-terminal of an adaptor domain N57 + SB100X (Kovac et al., 2020). N57 is the N-terminal 57 amino acids of the SB transposase with both DNA-binding and protein dimerization functions, which is used to recruit the Transposon sequence or/and SB100X *in vivo* (Izsvak et al., 2002). The result indicates that dCas9-SB100X and dCas9-N57 + SB100X show similar ability to target insertions in both single HPRT gene and multiple AluY, representing nearly 4% within 500bp.

In 2021, Pallarès-Masmitjà and her colleagues described another gene delivery tool (Find and cut-and-transfer, FiCAT) combining Cas9 with the PB transposase (Pallares-Masmitja et al., 2021). Unlike the previous two designs, this study demonstrates that Cas9 but not dCas9 produces better results in targeted and overall insertion. These results indicate that double-strand break (DSB) activity might be vital in facilitating targeted integration. This has been confirmed by using a zinc finger-PB transposase fusion (Znf-PB) with or without an independent Cas9 nuclease. Only Znf-PB + Cas9 + gRNA shows the highest percentage of GFP-positive cells (a reporter line). In addition, the authors demonstrate that the delivery efficiency of FiCAT is 4%–20% in human (HEK293T, K-562) and mouse (C2C12)) cells and *in vivo* in mouse liver. This competent design has achieved the on-target delivery efficiency of 25% after directed evolution (Pallares-Masmitja et al., 2021).

Compared to the classical CRISPR-Cas9 system, RNA-guided transposition systems provide an extensive external DNA delivery system to the mammalian cell genome. Compared to classical transposon techniques, RNA-guided transposition systems can provide an accurate delivery that will protect most essential genes or oncogenes in the genome. Another advantage is that RNA-guided transposition systems could restrict the total copy number of insertions, and classical transposon systems could not easily control it. As an example, transcriptome analysis of two cases of T cell lymphoma following PB-mediated CAR T cell therapy revealed significant copy-number alterations. These included widespread gains in oncogenes and losses in tumor-suppressor genes, all attributed to uncontrolled random insertions (Micklethwaite et al., 2021). By employing an RNA-guided transposition system, such an effect could be substantially mitigated or even eliminated.

#### 3.4.2 RNA-guided transposition system in nature

**FIGURE 9:**
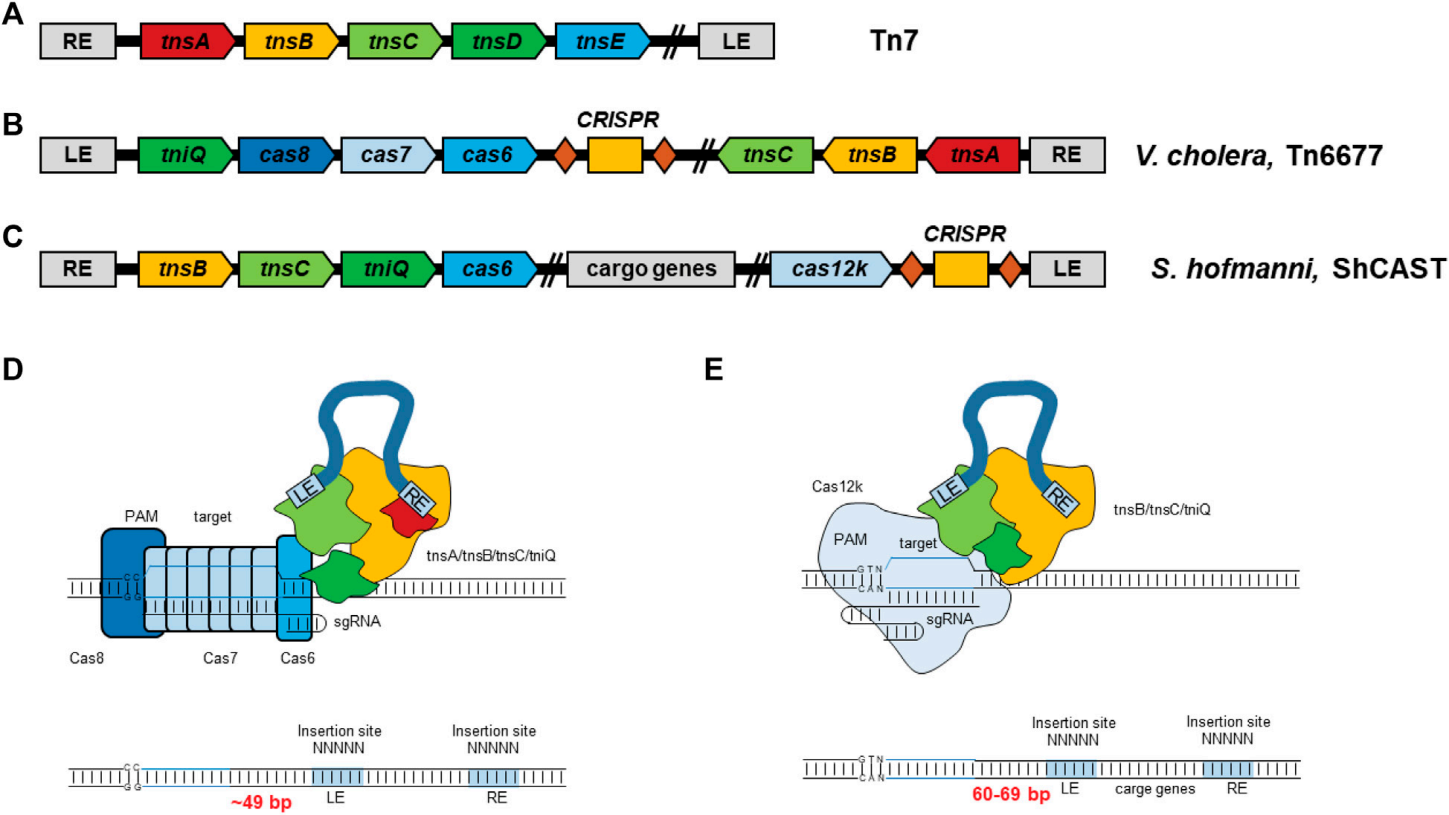
Schematic of RNA-guided DNA insertion with CRISPR-associated transposases. **(A)** Sequence structure of the classic Tn7 transposon; **(B)** Sequence structure of Tn6677 from *V. cholerae* strain HE-45; **(C)** Sequence structure of CRISPR-associated transposase and transposon from cyanobacteria *Scytonema hofmanni*; **(D)** Model for RNA-guided DNA transposition of Tn6677; **(E)** Model for RNA-guided DNA transposition of shCAST.

Meanwhile, bioinformatics analysis has identified several Tn7-like transposons that encode the CRISPR-Cas system, suggesting the existence of RNA-guided transposition (Peters et al., 2017; Faure et al., 2019). Classically, the Tn7 transposon encodes three essential proteins (two heteromeric transposases (TnsA and TnsB) and a regulator protein (TnsC)) to form the core transposition machinery, the TnsABC complex. In addition, Tn7 transposon encodes two target site-selection proteins, TnsD and TnsE. TnsD prefers to bind the “Tn7 attachment site,” *attTn7*, and then it will recruit TnsABC and donor DNA complex to integrate the transposon into the *attTn7* site. TnsE is a protein that prefers to bind the plasmid DNA, and then it recruits the TnsABC and donor DNA complex, integrating the donor at the target site. TniQ is a homolog of TnsD, which is incompletely characterized at the molecular level (Figure 9A).

Two groups have recently demonstrated two RNA-guided DNA insertion with transposon-encoded CRISPR–Cas systems: 1) type I-F CRISPR-Cas System (Klompe et al., 2019); and 2) type V-K CRISPR-Cas System (Strecker et al., 2019) (Figures 9B, C). Both systems are Tn7-like transposition machinery, including three key proteins: TnsA, TnsB, and TnsC, which together bind and cleave donor DNA through recognition of specific sequences but lacking TnsD/TnsE.

To test the hypothesis that a transposon-encoded CRISPR-Cas system could direct transposons to genomic sites through guided RNA, Klompe and colleagues studied the transposition mechanism of the type I-F CRISPR-Cas system from *Vibrio cholera* (Figures 9B, D). The elements of the type I-F CRISPR-Cas system were split into three separate plasmids in *Escherichia coli*, containing: 1) the core transposition element, TnsA-TnsB-TnsC; 2) the CRISPR machinery, TniQ-Cas8-Cas7-Cas6, along with a native CRISPR array encoding mature CRISPR RNAs (crRNAs); 3) the donor DNA. Transfection experiments confirmed that all these three plasmids are essential for RNA-directed transposition. Next, the authors attempted to knock out or to make mutants on each of the genes and found that all the encoding genes and crRNA are essential for this RNA-directed transposition.

Strecker and colleagues used a similar plasmid-based approach to demonstrate site-directed transposition with a single Cas effector using the type V-K system, which they named CAST (CRISPR-associated transposase) (Figures 9C, E). The authors designed a three-plasmid system from *cyanobacteria Scytonema hofmanni* CAST into *E. coli* system to analyze the transposition mechanism, including a helper plasmid containing TnsB, TnsC, TniQ, and Cas12k, along with the endogenous tracrRNA region and a crRNA; a donor plasmid containing the kanamycin resistance gene flanked by the transposon; and a target plasmid containing a synthetic protospacer sequence flanked by a short random motif upstream of the protospacer. Using this system, ShCAST was confirmed to catalyze RNA-guided DNA transposition by inserting DNA segments to 60–66 base pairs downstream of the protospacer unidirectionally with high efficiency in microbiome genomes.

Unfortunately, until now, transposon-encoded CRSIPR-Cas systems do not exhibit enough efficiency in eukaryotic cells (Lampe et al., 2023). In addition, further studies should engineer an easy-to-use system suitable for more species. However, the first steps have been very encouraging for synthetic biology and metabolic engineering. It also provides more information to design a more efficient CRISPR-transposon system based on the fully developed CRISPR-Cas9 system and PB or SB transposition system.

## 4 Conclusion

The studies above highlight the breadth of current research interests involving transposable elements. The mechanics of transposition and the dynamic interplay between transposable elements and their host organisms have been the central subjects of intensive investigation for an extended period. During evolution, transposable elements have played a critical role in forming the structures and regulatory elements of genomes in different species. A recent study showed that transposable elements could promote exon shuffling by inserting transposase domains in new genomic contexts. This mechanism can generate host-transposase fusion genes through alternative splicing and provides a believable path for the evolution of several ancient transcription factors with crucial developmental functions (Cosby et al., 2021). This finding is similar to the mechanism of antibody formation in the immune system. In individuals, transposable elements are usually kept repressed, and excessive activation of transposable elements can lead to several diseases. Transposable element overactivation leads to gene mutagenesis, structure changes and chromosome rearrangements in the genome, and the activation of the cGAS-STING inflammation pathway. A recent study shows that CRISPR–Cas9-mediated deletion of a LINE retroelement Lx9c11 in mice leads to the lethal immune response to virus infection through regulations of the Schlafen family genes, which indicates that there probably exist other unknown inflammation-related mechanisms and pathways for further validation.

Conversely, when examining the activity of particular transposable elements within distinct tissues of the organism, a different picture emerges. For instance, some transposable elements are not silenced in the brain, especially in the hippocampus. Some transposable elements mediate cell-to-cell interactions and DNA, RNA, or protein exchanges. These new findings indicate that the functions of transposable elements are extremely abundant. Transposable elements should be considered highly regulated rather than deeply repressed in the human body.

Secondly, transposable elements have served as indispensable tools for extensive phenotypic screening, leading to the discovery of numerous pivotal signaling pathways through this technology. Transposable elements have been instrumental in generating specific libraries for high-throughput sequencing, encompassing techniques like ATAC-seq for exploring open reading frames and LIANTI technology for the linear amplification of single-cell genomes. Retrotransposons, critical players in mechanism studies, have yet to be fully applied to the development of biological tools. In the near future, there is a growing inclination towards advancing retrotransposon-based tools. Recently, a new CRISPR-Transposon has been discovered with a novel mechanism of RNA-directed targeting of transposition, and a series of novel technologies have been developed based on a similar principle. This technique has a high potential to facilitate targeted DNA integration without the off-target mutagenesis potential of methods utilizing homologous recombination.

Additionally, another potential direction is that TEs harbor an abundance of native cis-regulatory sequences that allow special and temporal regulation of both upstream and downstream genes, guide copies, deletions, and recombination. These resources warrant further exploration and investigation (Palazzo and Marsano, 2021).

In addition, we should also note that mechanistic studies of TEs often go hand in hand with TE-based technology development. As a mutagen, TE plays a vital role in the occurrence of cancer. At the same time, the modified transposition system has also been employed to decode the essential genes involved in cancer development systematically. At the same time, the efficient mutagenicity of TEs can accelerate efforts to understand the mechanisms of cancer treatment and recurrence. In the context of brain cancer, recurrent tumors following surgical resection and radiotherapy exhibit substantial distinctions from the primary tumor. As a mutagen, TE is also critical in the occurrence of various genetic diseases. At the same time, the use of modified TE systems allows investigators to systematically analyze the key regulatory elements in individual growth and development. For example, Chang and colleagues developed a highly efficient forward genetic screening system in mice and discovered a critical gene that causes milk-feeding disorders in mice (Chang et al., 2019). Now, many new mechanisms related to TEs, such as their roles in inflammation and aging, have been deciphered, which indicates that TEs and their associated regulatory proteins may be candidate targets for anti-inflammatory and anti-aging therapies in the future. Technological advances could facilitate its associated techniques. With the development of high-throughput sequencing, not only DNA transposon but also retrotransposable elements could be employed as a power tool for genome-based modifications. Overall, the next few years will likely witness an expanding interest in transposon biology, leading to scientific advancement and the establishment of broad human health benefits.
